# Design and implementation of a mobile health electronic data capture platform that functions in fully-disconnected settings: a pilot study in rural Liberia

**DOI:** 10.1186/s12911-020-1059-6

**Published:** 2020-02-22

**Authors:** Avi Kenny, Nicholas Gordon, Jordan Downey, Owen Eddins, Kathleen Buchholz, Alvin Menyon, William Mansah

**Affiliations:** 1 Department of Biostatistics, University of Washington, 1705 NE Pacific Street F-600, Seattle, WA 98195 USA; 2 Last Mile Health, 205 Portland St #200, Boston, MA 02114 USA; 3 Liberia Ministry of Health, Tubman Blvd, Congo Town, Monrovia, Liberia

**Keywords:** Data collection, mHealth, eHealth, Digital health, Mobile health, Community health worker, Offline, Electronic data capture, EDC, Disconnected

## Abstract

**Background:**

Mobile phones and personal digital assistants have been used for data collection in developing world settings for over three decades, and have become increasingly common. However, the use of electronic data capture (EDC) through mobile phones is limited in many areas by inconsistent network connectivity and poor access to electricity, which thwart data transmission and device usage. This is the case in rural Liberia, where many health workers live and work in areas without any access to cellular connectivity or reliable power. Many existing EDC mobile software tools are built for occasionally-disconnected settings, allowing a user to collect data while out of range of a cell tower and transmit data to a central server when he/she regains a network connection. However, few tools exist that can be used indefinitely in fully-disconnected settings, where a user will never have access to the internet or a cell network. This led us to create and implement an EDC software tool that allows for completely offline data transfer and application updating.

**Results:**

We designed, pilot-tested, and scaled an open-source fork of Open Data Kit Collect (an Android application that can be used to create EDC systems) that allows for offline Bluetooth-based bidirectional data transfer, enabling a system in which permanently-offline users can collect data and receive application updates. We implemented this platform among a cohort of 317 community health workers and 28 supervisors in a remote area of rural Liberia with incomplete cellular connectivity and low access to power sources.

**Conclusions:**

Running a fully-offline EDC program that completely bypasses the cellular network was found to be feasible; the system is still running, over 4 years after the initial pilot program. The users of this program can theoretically collect data offline for months or years, assuming they receive hardware support when needed. Fully-offline EDC has applications in settings where cellular network coverage is poor, as well as in disaster relief settings in which portions of the communications infrastructure may be temporarily nonfunctional.

## Background

Mobile phones and personal digital assistants have been used for data collection in developing world settings for over three decades, and have become increasingly common [1–7]. Potential advantages of electronic methods over paper-based methods include lower error rates [3, 6], reduced likelihood of data loss [1], higher data completeness [2, 3, 6], reduced time needed for data collection [2, 3, 6, 8], feasibility of advanced data quality strategies [9], and in some cases decreased costs [2, 6, 10]. This class of techniques, known as *electronic data capture* (EDC) has been shown to be feasible among users with little or no prior experience with data collection or mobile phone use in a number of different settings, provided that they are given some basic training [1, 2, 5], and has been largely seen as acceptable by managers, users, and data collection subjects [2, 5, 6, 10, 11]. Additionally, the use of mobile phones may help to reinforce both clinical and non-clinical processes, leading to improved quality of care and greater efficiency [12, 13]. Thus, it represents an attractive option for researchers, governments, non-governmental organizations and others interested in large-scale data collection.

However, the use of EDC through mobile phones is limited in many areas by inconsistent network connectivity and poor access to electricity, which thwart data transmission and device usage. Many existing EDC mobile software tools are built for *occasionally-disconnected settings*, allowing a user to collect data while away from the cell network and transmit data to a central server when he/she has a network connection. However, few tools exist that can be used indefinitely in *fully-disconnected settings*, where a user will never have access to the internet or a cell network.

One setting in which these issues are quite common is rural Liberia, where much of the population lives in areas without access to cellular connectivity. Among remote communities of Rivercess County (those greater than five kilometers from the nearest health facility), 31.8% of the population lives in a community that does not have any cell network reception [14]. Additionally, there is no traditional power grid, and only 4.1% of Liberia’s rural population owns an electrical generator [15]. Rivercess is one implementation site of a national community health worker (CHW) program supported by the Liberia Ministry of Health and Last Mile Health (LMH), a non-governmental organization that works with government and other partners to design, demonstrate, scale, and advocate for national networks of professional CHWs. Because of the connectivity and power issues in Rivercess, program managers could not implement a traditional EDC system to collect data from CHWs; this necessitated the creation of a new tool that could function despite these infrastructural challenges.

The objective of this paper is to describe key features and lessons learned from the development and implementation of a fully-offline mobile phone EDC platform among a cohort of CHWs in a remote area of rural Liberia with incomplete cellular connectivity and low access to power sources. While some implementations of EDC software packages use offline data transfer as a backup mechanism, the system we describe is the first to be documented that intentionally bypasses the cellular network, instead using offline data transmission and application updating.

## Implementation

### Overview

We designed, pilot-tested, and scaled an open-source fork of Open Data Kit (ODK) Collect, an Android application that can be used to create EDC systems [16]. This fork is called “ODK-Liberia” and is freely available online as a licensed open-source application [17]. The application was initially implemented among a small pilot test group of seven CHWs and one supervisor. Later, the application was scaled to a cohort of 317 CHWs and 28 supervisors in remote areas of Liberia, representing the entire network health workers supported by LMH at the time. The overall objective of pursuing EDC technology was to increase the quality and efficiency of CHW-provided clinical care by increasing data quality, timeliness, completeness, and usage.

### Application development

Between February and April of 2015, ODK-Liberia was forked from the latest stable ODK Collect source code (v1.4.5) and developed. The primary functional addition was a Bluetooth-based data transfer system, which allows an end user to transmit data to another ODK-Liberia user in the absence of cell network or internet connectivity. This enabled the transfer of application updates (a set of ODK “blank forms”) and/or collected data (a set of ODK “completed forms”) from one user to another. From a technical perspective, this modification was simple. All blank forms in ODK Collect are stored as XML documents, conforming to the JavaRosa subset of the XForms 1.0 specification [18], in the *odk/forms* directory of the Android filesystem. Completed forms are stored as individual XML documents in the *odk/instances* directory. Our new feature allowed for these files to be transferred from the respective directories in the source device to those in the destination device. In the case of the application update, the contents of the source device are unchanged and the contents of the destination device are deleted and replaced by the new files. In the case of the transfer of collected data, files in the source device are moved to a new directory (*odk/archive*) in the same device (as a data backup mechanism) and copied into the *odk/instances* directory of the destination device. Data can be transferred any number of times between different Android devices.

Although this paper focuses on the Bluetooth transfer functionality, there were several secondary modifications made as part of the ODK-Liberia fork. One modification was a system that allowed for role-based access to forms, such that distinct user groups, such as CHWs and supervisors, would have access to different sets of forms. The many-to-many relationship between forms and roles is specified within a simple custom XML file which defines these associations. Any mobile device with ODK-Liberia installed can take on any role at any time; an administrator simply has to use a password-protected section of the user interface to change the value of a configuration variable. We also made several user interface modifications, including disallowing the deletion of completed forms and minor stylistic changes.

**Fig. 1:**
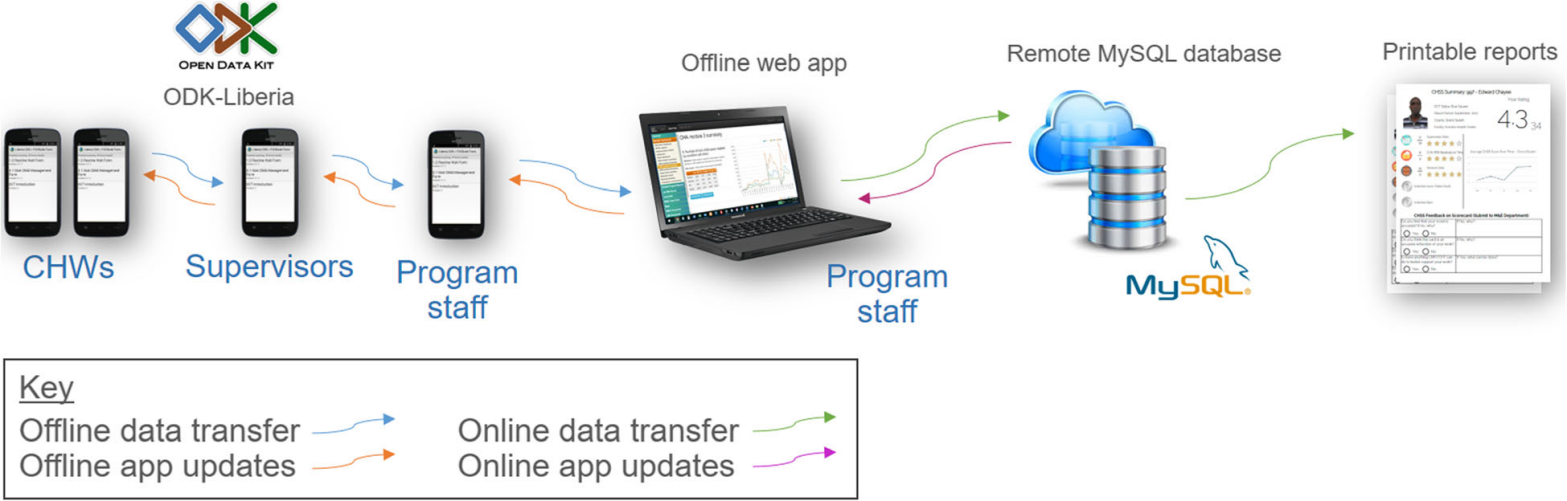
Data System Architecture

Additionally, we created an open-source Windows native application [19] to facilitate analogous data transfers between an Android device and a Windows computer. When receiving completed forms, the Windows application concatenates all forms with a custom delimiter in between and saves this as a single file with a custom file extension. This is done to facilitate easy upload into LMH’s custom web-based database application [20], which parses the data into JSON format, checks for file integrity, adds several metadata attributes, and sends the resulting dataset into a MySQL database cloud-hosted on a virtual private server. Note that the process described in this paragraph does not need to be replicated to take advantage of ODK-Liberia’s Bluetooth offline data transfer functionality; collected data could just as easily be sent to a ODK-compatible server, such as ODK Aggregate [21], once a user gains connectivity. A high-level snapshot of the overall data system architecture is given in Fig. 1 below.

**Fig. 2:**
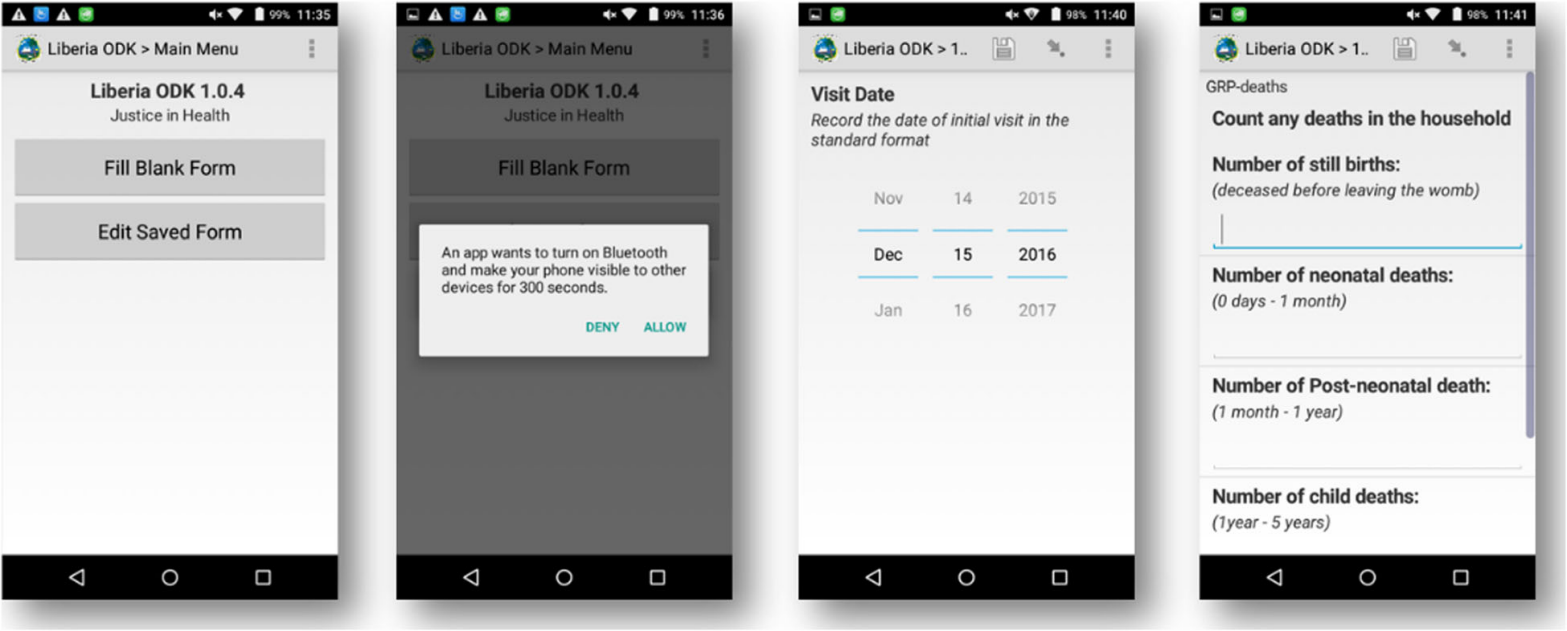
Select application screenshots

Forms were built using XLSForm and were designed to simultaneously function as both clinical decision support tools and data collection tools. For example, the “sick child form” supported the integrated community case management (iCCM) intervention, through which CHWs treated simple cases of malaria, diarrhea, and pneumonia at home and referred complicated cases to the nearest health facility. This form collects data, while guiding CHWs to arrive at the correct diagnosis and treatment for a particular disease. Other forms similarly rely on the use of automated skip logic, pre-programmed clinical algorithms, and form validation to help guide CHW workflows and provide individual decision support. Several screenshots of the application are shown for illustrative purposes in Fig. 2 below.

### Equipment

We chose to use BLU Product’s mobile devices for our EDC system based on a comparison of advertised battery life, durability, and price-point among a selection of Android devices. We primarily used the BLU Advance 4.0 model, which has a 4-in. 480 × 800 pixel display, a 1600 mAh battery, 4GB of internal storage space, and 512 MB of RAM. However, following the discontinuation of the Advance 4.0 model, we have also tested and implemented a variety of other BLU phones. To help prevent against damage during Liberia’s seven-month rainy season, we also procured waterproof cases, which allow for the device to be used while it is inside the case (through a touch-sensitive clear plastic front panel). In addition to these items, we equipped each CHW with an Anker 15 W Solar Panel and an Anker 15,600mAh battery pack, as no power grid or generators are available in many of the communities served by the program. CHWs were instructed to use the solar panel to charge the battery pack during the day, and then use the battery pack to charge the mobile phone in the evening. They were also given detailed instructions on how to prevent damaging the device (not using it in the rain, not giving it to children to play with, etc.). At times, we also employed a battery exchange process (particularly during the rainy season), in which supervisors would swap out depleted battery packs with fully-charged replacements, and then charge the depleted packs once they reached a power source (typically a generator). To reduce battery drain and unintended device usage, we installed custom-built kiosk software (source code will be made available soon) on each device, which restricted users from accessing any Android functionalities other than a small set of specified applications. The total initial equipment cost was $123 per CHW (excluding shipping costs), and we have observed that each piece of equipment usually lasts roughly 2–3 years. Shipping was provided as an in-kind donation; actual shipping costs could vary widely depending on location and program scale and should always be accounted for.

### Pilot phase

We tested the EDC platform during an initial pilot phase, which lasted from February 5th to April 9th, 2015. The initial pilot phase included seven CHWs and one supervisor, selected purposively because of their close physical proximity to one of our field site offices. Participants were given a two-day training on EDC tools and processes, which included an overview of equipment usage, mock clinical scenarios, and form practice. Participants were given field manuals, which provided graphics on how to use different features of the EDC application such as touching, swiping, and putting devices in sleep mode. Two forms were used for the pilot, the sick child form described above and a form used to screen patients for Ebola, as active CHW surveillance for Ebola symptoms was still ongoing at the time.

To evaluate the pilot phase, we conducted post-training and a post-implementation focus group of all training participants and reviewed unstructured field notes taken by implementers. The focus group lasted a full day, and involved asking participants open-ended questions around overall successes and challenges, as well as directed questions around software usability, hardware challenges, community member reactions to the program, and perceptions of the pilot (see Appendix 1 for the focus group questionnaire).

### Scale-up phase

The scale-up phase lasted from July 1st, 2015 until December 15th, 2016; however, the program is still fully operational as of December 2019. For this phase, training lasted roughly 12 h and was embedded into existing programmatic training modules. Topics covered and materials used were similar to those covered in the pilot phase. The current training materials are available as an Appendix. CHWs were equipped with a redesigned version of the iCCM form, as well as a form to capture data on routine monthly home visits. The supervisor forms captured information on supervision activities and supply chain. Notably, the supervision form included both a geo-tag and a timestamp, which helped to prevent data falsification. During biweekly supervision visits, supervisors were responsible for transferring all CHW-collected data to their mobile phone, as well as transferring any updates to the CHW’s phone. When the supervisors returned to the central office for meetings and stock refills, they would in turn transfer their data to the phone of a staff member on the LMH monitoring & evaluation team. In this sense, the flow of data paralleled the flow of physical commodities, such as medications. The LMH staff member would then transfer data to their laptop and upload it to the LMH database.

To evaluate the scale-up phase, we conducted a number of semi-structured interviews of both CHWs and supervisors, reviewed metrics of data quality and completeness, and reviewed field notes taken by implementers.

## Results

### Pilot phase

The pilot phase (2/5/2015–4/9/2015) involved seven CHWs and one supervisor in Grand Gedeh County. Of the initial piloted participants, only one CHW had ever used a smartphone. Of the three CHWs that reported owning non-touch screen mobile phones, all reported that their community had mobile network access.

Participants reported that it took one to 3 weeks of field-based use to become comfortable with the use of the phones and application. Of note, all EDC tools were modeled after a paper-based equivalent, on which the CHWs were already trained; this likely contributed to the ease with which participating CHWs were able to learn. Some CHWs requested for more advanced functionality to be added, such as automatic populating of form fields and longitudinal patient visit records. When asked to compare paper forms to EDC, CHWs reported that EDC was easier to use, decreased time spent writing, and lightened the set of materials that needed to be carried around during patient visits. One CHW noted, “I never get a form [sent back] to me with an error”. They also mentioned that it improved their perceived status in their communities and that they felt that their capacity was being built to learn new technologies. When asked to identify weaknesses of the EDC platform, CHWs reported specific application bugs in the tools, as well as the lack of more advanced application functionality, including the ability to dynamically access previously-entered data. When asked about community perceptions of the system, one CHW noted, “It [provides a great morale boost] for patients to be treated by [a health worker with] a computer”.

The solar charging scheme proved to be effective. There was not a single reported instance of a CHW being unable to complete a form because of lack of power. However, the pilot was conducted during Liberia’s dry season, and as noted below, we experienced charging difficulties due to equipment malfunction once the program scaled up, especially during rainy season.

Due to the technical feasibility and enthusiastic acceptance by CHWs, the pilot phase was generally viewed internally as a success and paved the path for the subsequent scaling of the program.

### Scale-up phase

The scale-up phase (7/1/2015–12/15/2016) involved 317 CHWs and 28 supervisors across Rivercess and Grand Gedeh Counties. During this period, 63,092 individual forms were submitted, including 22,824 iCCM forms, 36,978 routine visit forms, 1420 supervision forms, and 1870 restock forms.

We also found that many CHWs would conduct client visits without their mobile device. They would then retroactively complete the forms during the evening, either from written notes or from memory. We initially identified this through field observations, and subsequently through an analysis of the timestamps automatically taken at the start and end of each form, noticing that batches of forms would often be filled out at the end of the day by a CHW. Within the time period analyzed, the median time between forms (excluding the time between the last form of a given day to the first form of the next day) was 10 min, which implies that the majority of CHWs were simply using the phones for retroactive “data entry” rather than using them as decision-support tools during the actual patient interaction. This finding led to field-based retraining of CHWs to encourage use of the phones as intended.

The median length of time it took for a routine visit form to reach the database (calculated as the database INSERT timestamp minus the form completion timestamp) was 24 days (IQR: 17 days), and the median time for an iCCM form was 27 days (IQR: 22 days). The median time for a supervision form to reach the database was just 17 days (IQR: 19 days), which can be explained by the fact that the data generation for this form is happening “one step upstream” of the CHW-generated data, and was thus able to reach the database in less time. While not ideal, these delay lengths were considered acceptable, in part because the biweekly frequency of supervision placed a limit on how quickly data could be collected in the field and in part because the data captured through this system was routinely used the following month (rather than the current month) by program managers.

The main issue that plagued the scale-up phase was device malfunction. Because of ongoing delays in procurement (caused by various issues, such as the difficulties associated with shipping large quantities of devices powered by lithium ion batteries), many CHWs remained without one or more pieces of equipment for months at a time. Often, when the solar charger, power bank, or USB cable was broken, CHWs utilized local generator-powered commercial charging booths. Unfortunately, we did not collect detailed data on device malfunction rates or accessibility of alternative power sources.

## Discussion

Overall, we found that ODK-Liberia was usable and acceptable to CHWs, and served as an effective technical solution for the connectivity issues. This was not surprising, given previous feasibility assessments of EDC [1, 2, 5]. Data generated through the EDC program is now used regularly within various data reports and tools as part of their routine programmatic monitoring system. These tools include reports on under-five child treatment, form completion, supervision performance, and routine visit activities, disaggregated at various levels (e.g. by county, by district, or by CHW).

The primary advantage of ODK-Liberia over the majority of existing EDC platforms is that transfer of both data and application updates (i.e. blank forms) can happen in the complete absence of connectivity. This allows for data collection to occur indefinitely among user groups who have no access to a cell network or the internet for long periods of time (on the order of months or years). This functionality enabled the implementation of an EDC system which has been up and running for over 4 years, eventually among 317 CHWs and 28 supervisors. The most widely-used EDC packages either do not have this functionality, can only do one-way offline data transfers from the users to the server, and/or have functionality that requires a skilled technician (e.g. using a laptop to update a phone) and may be prone to human error. Additionally, although it is not currently used in this particular implementation, ODK-Liberia maintains its native data exchange capabilities, so if connectivity is present or regained, data can be transmitted over the cell network or internet. Thus, even though our implementation collected and transferred 100% of the data offline, it is very much possible for “hybrid” implementations to be deployed in which some users can submit data and receive updates over the cell network and others can do so offline. Similarly, individual users can utilize both data transfer mechanisms depending on what is the most feasible at any given point in time.

It should be stressed that ODK-Liberia was a tool created for the specific needs of the Liberia CHW program. Although the software is open-source, stable, and usable by anyone, the authors recommend that the creators of widely-used EDC platforms develop and implement similar functionality within their own tools.

We see two general use cases for this functionality. The first use case is data collection within settings that have similar connectivity issues to those of rural Liberia. Any group who wants to run an EDC program but has struggled to do so because of connectivity constraints can do so with ODK-Liberia or with software that contains similar functionality. The second is during disaster relief efforts. Natural disasters often cause severe damage to existing communications infrastructure [22, 23], which may make the use of a traditional EDC system impossible. Data collection during natural disasters can be extremely challenging for a variety of economic, political, and technical reasons [24], and since having strong data collection systems can lead to more effective and coordinated responses and relief efforts [25], an EDC system with the offline-transfer functionality of ODK-Liberia could play a vital role.

With both use cases, offline data transfer may be far cheaper than existing alternatives such as satellite-based data transfer, and possibly cheaper than paper-based alternatives when the costs of data quality assurance, aggregation, and reporting are taken into account. Because of potential costs savings, a fully-offline data system should not be viewed only as a backup mechanism, but as a potential option for the main means of data collection. With a fully-offline system, there is no need to purchase SIM cards or cellular credit, resulting in immediate savings. Furthermore, given that an often-recommended solution for EDC platform users who live in disconnected areas is to travel to another community to send their data to a server via the cell network, a fully-offline implementation may result in savings in terms of staff time and travel costs. Projected cost savings is the primary reason that the Liberia CHW program uses offline transfer as the sole mechanism for data exchange, rather than as a backup mechanism, even for CHWs who live in communities with reliable cell network. This being said, the costs of equipment, training, and staff time were substantial, (especially relative to the per capita GDP of a country like Liberia) and any government or organization considering implementing an EDC program should conduct a comprehensive cost-benefit analysis to assess whether it is the correct decision.

There are limitations to both the software tested and to this pilot study. The main limitation of a fully-offline data transfer workflow is that there can be substantial delays between when the data is generated and when it hits a central database. This makes this sort of system insufficient for data capture programs in which such a delay is unacceptable, such as an infectious disease outbreak surveillance system. Other limitations include the need to manage and monitor data completeness within a more complex data transfer pathway, greater potential for human error, and the potential for technically-savvy “upstream” users to tamper with the data. Human error can potentially be decreased through additional automation of the bidirectional data transfer process, and data tampering, while not a realistic concern in our setting, could be mitigated through software changes that would encrypt underlying data and disallow editing by secondary users. Limitations of this pilot study include the lack of a formal evaluation, restriction to a single country and implementer, and restriction to the single use case of data collection within a community health worker program. Additionally, we did not collect pre-implementation data or data from a control group on quantitative indicators of data quality, such as completeness or accuracy; this would have allowed us to better understand the benefits and drawbacks of the EDC system as compared to the previous paper-based system.

## Conclusions

Running an electronic data capture program that bypasses the cellular network was found to be feasible. The users of this program can work offline indefinitely, assuming they receive hardware support when needed. Fully-offline EDC has applications in settings where the density of cellular towers is low, as well as in disaster relief settings in which portions of the communications infrastructure may be temporarily nonfunctional.

## Availability and requirements

**Project name**: ODK-Liberia.

**Project home page**: https://github.com/Last-Mile-Health/ODK-Liberia

**Operating system(s)**: Android OS 7.0 or higher.

**Programming language**: Java.

**Other requirements**: none.

**License**: Apache License 2.0.

**Any restrictions to use by non-academics**: none.

## Supplementary information

**Additional file 1.** Feasibility Assessment questionnaire.

## Data Availability

The datasets used and/or analyzed during the current study available from the corresponding author on reasonable request.

## Abbreviations

CHW: Community health worker
EDC: Electronic data capture
iCCM: integrated community case management
LMH: Last Mile Health
ODK: Open Data Kit
