# Supplementary material for: Design and implementation of a mobile health electronic data capture platform that functions in fully-disconnected settings: a pilot study in rural Liberia
Source: BMC Med Inform Decis Mak. 2020 Feb 22;20:39. doi: 10.1186/s12911-020-1059-6 (PMC7036217; doi:10.1186/s12911-020-1059-6)
Supplement: Supplementary file 1 — Additional file 1. Feasibility Assessment questionnaire. [file 12911_2020_1059_MOESM1_ESM.docx]

**Appendix 1: Feasibility Assessment questionnaire**

General

1. Let’s look at the PROs of mHealth. Feel free to let us know anything that you feel about the ways that mHealth was helpful for you and for your work.
2. Now I want to look at the CONs of mHealth. Please tell us anything that you feel was not working well.
3. For you personally, do you feel that you have learned any new skills?
4. Exercise: Do two sample forms together and observe.

mHealth Usability

1. When filling out the forms in mHealth and on paper, did you start with using the data collection tool (DCT) or did you start with using the paper forms?
2. Which do you think is easier, mHealth or the paper forms?
3. Are there any other things that you liked about using the DCT?
4. Were there any other things that you did not like about using the DCT?
5. Now that you have used mHealth for two months, how you feel about using mHealth for all your forms?
6. When we had the training, some of you were still getting used to mHealth and the DCT. Do you feel like you have mastered the DCT and mHealth?
   1. If so, when did you become comfortable using the technology?
7. Do you think that you could help teach other CHWs how to use the DCT?

Hardware

1. Can you tell me of a time when your DCT did not work for you? For example, things like the DCT not turning on or not saving the forms.
   1. Were you ever unable to complete a form because of this problem?
2. Can you tell me of a time when you ran out of current on your battery pack?
   1. This time when you ran out of current, was it because of the battery pack or the solar panel?
3. When you were charging the battery pack, where did you leave the solar panel? Did you leave it at your home? At a friend’s home?
4. When the rain is coming, how did you keep your DCT safe from the water?
5. Now, since not everyone received the same DCT, I want us to talk to each other about which DCT is the best.
6. Not everyone has the same battery or solar charger either. Just like before, I want us to talk about which battery pack and solar charger is the best.

Community Perceptions

1. What were other people thinking about the DCTs?
2. Did you face some pressure from people in the community to touch and play with your DCT?
3. Did you ever worry that someone might steal it?
4. Do you feel that people gave you more respect because you were using technology to help treat them?
5. Can you tell me about any time that people were afraid of the technology? Did they ever fear that the technology could do bad things to their health or their privacy?

Concluding Questions

1. What could we do to make mHealth easier for you to use?
2. Do you have any ideas of new ways we could help our patients with mHealth?
3. Do you have any other thoughts that you want to share before we finish? These can be about anything.
